# The Antimicrobial Effect of *Cornus mas* L. and *Sorbus aucuparia* L. Fruit Extracts against Resistant Uropathogens in Correlation with the Prevalence of Urinary Tract Infections in Companion Animals

**DOI:** 10.3390/ph17060814

**Published:** 2024-06-20

**Authors:** Mara Aurori, Cristiana Ștefania Novac, George Cosmin Nadăș, Smaranda Crăciun, Nicodim Fiţ, Sanda Andrei

**Affiliations:** 1 Department of Preclinical Sciences, Faculty of Veterinary Medicine, University of Agricultural Sciences and Veterinary Medicine, 400372 Cluj-Napoca, Romania; mara.aurori@usamvcluj.ro; 2 Department of Microbiology, Immunology and Epidemiology, Faculty of Veterinary Medicine, University of Agricultural Sciences and Veterinary Medicine, 400372 Cluj-Napoca, Romania; cristiana.novac@usamvcluj.ro (C.Ș.N.); gnadas@usamvcluj.ro (G.C.N.); smaranda.craciun@student.usamvcluj.ro (S.C.); nfit@usamvcluj.ro (N.F.)

**Keywords:** cornelian cherry, rowanberry, fruit extracts, UITs in companion animals, antimicrobial resistance

## Abstract

Urinary tract infections (UTIs) are a widespread condition in pets, with many antibiotics being prescribed, contributing to the rise in antimicrobial resistance, which is a worldwide threat. This study’s main objective was to analyze the in vitro antimicrobial activity of *Cornus mas* and *Sorbus aucuparia* fruit hydro-ethanolic extracts towards bacteria identified in the urine of companion animals experiencing UTIs. Urine samples were collected from dogs and cats (*n* = 83; 47 negative, 36 positive); several bacterial strains were identified (*n* = 49) belonging to the *Escherichia*, *Enterococcus*, *Staphylococcus*, *Proteus*, *Klebsiella*, *Enterobacter*, *Pseudomonas*, *Acinetobacter*, *Leclercia*, and *Kocuria* genera. Bacterial susceptibility was tested using the disk diffusion method, with the majority being resistant to several beta-lactams, quinolones, trimethoprim/sulfamethoxazole, and nitrofurantoin. Subsequently, 13 resistant isolates were selected to evaluate the fruits extracts’ antimicrobial potential using the agar well diffusion and broth microdilution methods. *Cornus mas* exhibited the greatest activity against Gram-negatives (primarily *Pseudomonas luteola*), while *Sorbus aucuparia* showed maximum effects towards Gram-positives (particularly *Enterococcus faecalis*). The MIC was 0.01 μg/μL for both extracts; the MBC was 0.08 μg/μL for *Cornus mas* and 0.05 μg/μL for *Sorbus aucuparia*. However, *Cornus mas* showed a stronger bactericidal effect. This is the first study to investigate these fruit extracts in UTI isolates of companion animals, and these extracts might be used as substitutes or adjuvants for antibiotics, thus contributing to a reduction in antimicrobial resistance.

## 1. Introduction

Since their discovery, antimicrobials have proven to be valuable medications for the treatment of human and animal bacterial infections. Their increased availability in poor nations has contributed to the modern-day rise of antimicrobial usage [1]. Regretfully, bacteria have always adapted to withstand the overuse of antimicrobials, a phenomenon that is known as antimicrobial resistance (AMR) [2]. This process is acknowledged as one of the most alarming and pervasive public health problems around the globe [3]. A contributing factor to this process might be the increasing pet population in European countries, where over 60 million cats and dogs coexist with humans in shared habitats [4]. This might result in a higher probability of unsuccessful animal therapies as well as the widespread use of human antibiotics in veterinary medicine, which could promote zoonotic AMR transmission. Unfortunately, evidence about the transmission of AMR bacteria by companion animals is currently scarce [3,5].

Bacterial urinary tract infections (UTIs) are estimated to be one of the three most common reasons for antibiotic usage in veterinary medicine, being responsible for 12% of total antibiotic prescriptions, as they promote the spread of multidrug-resistant bacteria [6,7]. This condition provides a therapeutic dilemma in the field of veterinary medicine because the use of antimicrobials in UTIs is extremely necessary [8]. Additionally, the extra-label use of antimicrobials, specifically human-only antibiotics, is exceedingly common in dogs and cats. The prescribing and consumption of crucial antimicrobials—aminoglycosides, third-generation cephalosporins, fluoroquinolones, and carbapenems—in companion animals is especially alarming, as it may pose an important threat to human health [9,10]. To limit their consumption, a microbiological exam and an antibiogram must be performed [7]. However, in order to ease the patient’s pain, empirical therapy is typically advised while test results are being processed; this can result in undesirable patient outcomes and may aid in resistance selection [11]. 

Nevertheless, germ resistance, several adverse effects, high dosages, and the poor effectiveness of conventional antibiotics have driven scientists to seek out herbal remedies for UTI treatments, as this field has been poorly studied in companion animal medicine [12,13]. Medicinal plants may constitute an excellent choice for the ongoing management of UTIs, especially when used in conjunction with prescribed antibiotics, because they possess antimicrobial properties and may potentially reduce the negative effects of antibiotics. Cranberries are believed to be the most efficient herbal remedy for mitigating UTIs; however, there are other promising medicinal plants for UTI treatment that have received less attention [14]. 

A potential antibacterial cure could be *Cornus mas* L., a member of the *Cornaceae* family, which has a large distribution in Europe’s southeast and the Caucasus region [15]. *Cornus mas* L. has been commonly utilized in traditional medicine due to its abundance of bioactive compounds. The primary constituents of these fruits are phenolic compounds, notably anthocyanins, phenolic acids, iridoids, and flavonoids [16]. It has also been shown that these berries possess a variety of biological qualities, the most important being antiradical, antiproliferative, anti-inflammatory, anti-hyperglycemic, anticoagulant, and hypolipidemic properties [17]. Additionally, one of the most essential characteristics of these fruits is their antimicrobial activity. As such, multiple *Cornus mas* L. fruit extracts have been investigated in a number of in vitro studies against bacterial reference strains. The results indicated significant antimicrobial activity against strains of *Staphylococcus aureus*, *Staphylococcus epidermidis*, *Streptococcus pyogenes*, and *Pseudomonas aeruginosa* [18,19,20], while other extracts demonstrated moderate activity against *Escherichia coli* [21]. 

*Sorbus aucuparia* L. is another medicinal plant historically used for its reputed high nutritional value [22]. It belongs to the *Rosaceae* family and grows predominantly in Europe [23]. The principal chemical compounds identified in *Sorbus aucuparia* L. fruits are polyphenols, which are mostly composed of proanthocyanidins, chlorogenic, and neochlorogenic acids [22]. Although there hasn’t been much research on *Sorbus aucuparia* L.’s biological activities, recent studies have been centered on its antioxidant [23,24,25], antiproliferative, and digestive effects [23,26,27], with particular attention on its cardiovascular and anti-diabetic effects [28,29,30,31]. Aside from these benefits, one vital attribute of these fruits is their antimicrobial action. Therefore, *Sorbus aucuparia* L. berries have proven to exert in vitro antimicrobial efficacy against some Gram-positive bacterial reference strains (different *S. aureus* strains and *Enterococcus faecalis*) [32], as well as several Gram-negatives (*E. coli* and *P. aeruginosa*) [22]. Additionally, a relatively mild action against *Klebsiella pneumoniae* by these berries was demonstrated [33].

Both *Cornus mas* L. and *Sorbus aucuparia* L. possess high quantities of polyphenols. It is well recognized that natural polyphenols are effective against bacterial infections, mainly due to their chemical makeup (aromatic structure and hydroxyl groups). The published research suggests that the primary mechanisms underlying the antimicrobial activities of these chemicals could imply bacterial membrane damage, alterations in permeability, polarization, and efflux pump inhibition. Additionally, polyphenols have been shown to alter gene functions linked to specific bacterial characteristics, such as hydrophobicity, adherence, flexibility, assault, and biofilm development [34,35].

In light of the requirement for novel treatment based on therapeutic plants that might counteract antimicrobial resistance and offer an alternative to human antibiotics in veterinary medicine, we decided to investigate the antimicrobial activity of *Cornus mas* L. and *Sorbus aucuparia* L. fruits, which were discovered to be valuable reservoirs of bioactive compounds displaying various biological properties. Moreover, considering that both fruits possess significant amounts of polyphenolic compounds and have demonstrated in vitro antimicrobial activity against several reference bacterial strains, we thought it would be worthwhile to thoroughly assess their antimicrobial activity against certain clinical isolates. Thus, the primary goal of this study was to assess the in vitro antimicrobial activity of hydro-ethanolic extracts derived from *Cornus mas* L. and *Sorbus aucuparia* L. fruits on pathogens isolated from the urine of companion animals diagnosed with urinary tract infections.

## 2. Results

### 2.1. Study Population

The study comprised 36 animals who had been diagnosed with a UTI; 20 subjects were dogs (55.56%), while 16 were cats (44.44%). A higher percentage of males was recorded in comparison to females (55.56% vs. 44.44%). In terms of age, UTI was predominantly diagnosed in young animals (14; 45.16%), followed by adults (13; 41.94%), and later in the geriatric population (4; 12.9%). However, five animals’ ages were unknown. Moreover, regarding the variety, 21 animals (58.33%) consisted of mixed breeds, while 15 (41.67%) were purebred. Furthermore, 10 individuals (27.78%) of the animal population had previously received antibiotic treatment, whereas 26 subjects (72.22%) had not experienced any antimicrobial therapy. Eleven animals (6 cats and 5 dogs) showed signs of coinfections with two or more bacterial strains, whereas 3 animals (2 dogs and 1 cat) were confirmed with recurrent UTIs (rUTIs). Specifically, these three subjects experienced both recurrent UTIs and bacterial coinfections. Table 1 summarizes all of the study population’s data.

### 2.2. Bacterial Isolates

Vitek^®^’s identification of clinical isolates revealed bacteria belonging to 10 distinct genera: *Escherichia*, *Enterococcus*, *Proteus*, *Klebsiella*, *Staphylococcus*, *Acinetobacter*, *Enterobacter*, *Kocuria*, *Pseudomonas*, and *Leclercia*.

The distribution of each isolated bacterial strain in dogs and cats is listed in Table 2. Forty-nine isolates in total—21 (42.86%) Gram-positive and 28 (57.14%) Gram-negative—were obtained from urine samples; the proportions of Gram-positive and Gram-negative bacteria in dogs and cats differed significantly (*p* = 0.031). Gram-negative bacteria were particularly abundant in canines, whereas Gram-positive isolates were more common in felines. Specifically, 7 Gram-positive and 18 Gram-negative bacteria were obtained from dogs, while 14 Gram-positive and 10 Gram-negative bacteria were found in cats.

*Escherichia coli* was the most frequently isolated bacteria, being the most prevalent pathogen in cats and the second most common in dogs; its incidence varied considerably between both species (*p* = 0.008). *Proteus mirabilis*, which was not seen in cats, was the most commonly recovered bacteria from dogs. Another significant pathogen present in dogs and absent in cats was *Klebsiella pneumoniae*. Furthermore, *Enterococcus faecalis* was the second most commonly identified bacteria, displaying a noteworthy variation in frequency between canines and felines (*p* = 0.041). Additionally, no statistical differences in *Enterococcus faecium* (*p* = 0.113), *Acinetobacter baumannii* (*p* = 0.317), and *Staphylococcus pseudintermedius* (*p* = 0.317) prevalence were registered between dogs and cats. Other dogs-only detected bacteria were *Enterobacter cloacae*, *Klebsiella oxytoca*, *Staphylococcus aureus*, *Pseudomonas luteola*, and *Kocuria* spp., whereas *Staphylococcus lentus*, *Staphylococcus equorum*, *Staphylococcus sciuri*, *Staphylococcus haemolyticus*, and *Leclercia adecarboxylata* were reported only in cats.

Of particular interest, one strain of *Escherichia coli*, *Enterococcus faecalis*, and *Klebsiella pneumoniae* were consistently discovered in samples processed from certain studied animal, being involved in the emergence of recurrent UTIs (rUTIs). In addition, *Staphylococcus equorum*, *Staphylococcus sciuri*, and *Leclercia adecarboxylata* were isolated from the same feline patient, contributing to the development of a complicated UTI. Moreover, both *Staphylococcus lentus* isolates were detected in samples where other bacteria were present.

### 2.3. Antibiotic Resistance of Clinical Isolates

Antimicrobial sensitivity findings showed that 28 (57.14%) isolates were resistant to at least one antibiotic, whereas 18 (36.73%) showed resistance to at least one agent from three or more classes of antimicrobials, which were categorized as multidrug-resistant (MDR) [36]. Particularly, it was found that dog samples displayed 14 resistant and 8 MDR strains, whereas cat samples contained 14 resistant and 10 MDR bacteria.

As such, most of the *E. coli* strains showed 60% resistance to clavulanate amoxicillin and cephalexin and 80% to marbofloxacin, enrofloxacin, nitrofurantoin, and trimethoprim/sulfamethoxazole. All *E. coli* isolates were resistant to cefuroxime and ciprofloxacin (100%). However, only a few bacteria were resistant to gentamicin (40%).

The *Enterococcus* group registered complete resistance to clavulanate amoxicillin and cefotaxime (100%). The majority of the strains showed resistance to cephalexin, marbofloxacin, enrofloxacin, ciprofloxacin, nitrofurantoin, gentamicin, and ceftriaxone (66.67%). Additionally, 33.33% of isolates were resistant to trimethoprim/sulfamethoxazole. 

Regarding *Proteus* spp., the clinical isolates were 100% resistant to cephalexin, trimethoprim/sulfamethoxazole, doxycycline, marbofloxacin, and nitrofurantoin and mostly resistant to clavulanate amoxicillin, enrofloxacin, ciprofloxacin, and cefuroxime (71.43%). Furthermore, 57.14% of bacteria showed resistance against gentamicin, while only 28.57% showed resistance to cefotaxime.

*Klebsiella* spp. was 100% resistant to clavulanate amoxicillin, cephalexin, cefotaxime, ceftriaxone, cefuroxime, trimethoprim/sulfamethoxazole, doxycycline, marbofloxacin, enrofloxacin, and nitrofurantoin. Additionally, 66.67% of isolates were resistant to gentamicin, and only 33.33% were resistant to ciprofloxacin. 

The *Staphylococcus* group registered resistance against trimethoprim/sulfamethoxazole, gentamicin, and enrofloxacin (50%).

*Acinetobacter baumannii* recorded resistance to clavulanate amoxicillin, cephalexin, cefuroxime, trimethoprim/sulfamethoxazole, gentamicin, enrofloxacin, and nitrofurantoin. Similar results were observed for *Enterobacter cloacae*; however, resistance to cefotaxime rather than cefuroxime was identified. Additionally, *Pseudomonas luteola* was resistant only to cefuroxime and nitrofurantoin, while *Kocuria* spp. showed resistance in a similar manner to *Staphylococcus* spp.

In terms of feline bacteria, complete resistance to clavulanate amoxicillin, cefotaxime, and ceftriaxone was observed in all *E. coli* strains. A great number of strains registered resistance to cephalexin (87.5%), nitrofurantoin (75%), enrofloxacin, ciprofloxacin, gentamicin, and cefuroxime (62.5%). Additionally, 50% of isolates were resistant to marbofloxacin and trimethoprim/sulfamethoxazole, while 25% were resistant to doxycycline.

*Enterococcus* spp. exhibited 87.5% resistance to clavulanate amoxicillin, nitrofurantoin, gentamicin, cefuroxime, and cephalexin. Most of the strains resisted the antimicrobial activity of cefotaxime (75%), marbofloxacin, enrofloxacin, and trimethoprim/sulfamethoxazole (62.5%). Furthermore, 50% of isolates registered resistance to ceftriaxone, and 37.5% to doxycycline. A small number of *Enterococcus* strains showed ciprofloxacin resistance (25%).

Regarding staphylococci, 100% resistance was registered to clavulanate amoxicillin, cefotaxime, cefuroxime, and trimethoprim/sulfamethoxazole. Additionally, 83.33% of isolates were resistant to cephalexin, gentamicin, enrofloxacin, and nitrofurantoin, while 50% were resistant to ceftriaxone, doxycycline, marbofloxacin, and ciprofloxacin.

*Acinetobacter baumannii* was resistant to clavulanate amoxicillin, cephalexin, cefotaxime, trimethoprim/sulfamethoxazole, gentamicin, enrofloxacin, and nitrofurantoin, while *Leclercia adecarboxylata* produced comparable results, but did not demonstrate resistance to cefotaxime.

Overall, the most effective antibiotic was doxycycline, while clavulanate amoxicillin exhibited the lowest antimicrobial activity. Specifically, in canine isolates, doxycycline displayed antimicrobial activity against all clinical isolates except *Proteus* spp. and *Klebsiella* spp. These bacteria were particularly sensitive to cefotaxime, ceftriaxone, and gentamicin. A weak antimicrobial activity was determined by clavulanate amoxicillin, nitrofurantoin, and trimethoprim/sulfamethoxazole. Feline isolates were more resistant compared to canine bacteria. Thus, doxycycline showed effectiveness against nearly half of the pathogens. Marbofloxacin and ciprofloxacin yielded slightly lower outcomes. Apart from the minimal impact of clavulanate amoxicillin, cefotaxime and trimethoprim/sulfamethoxazole demonstrated low antimicrobial activity towards feline bacteria.

**Figure 1:**
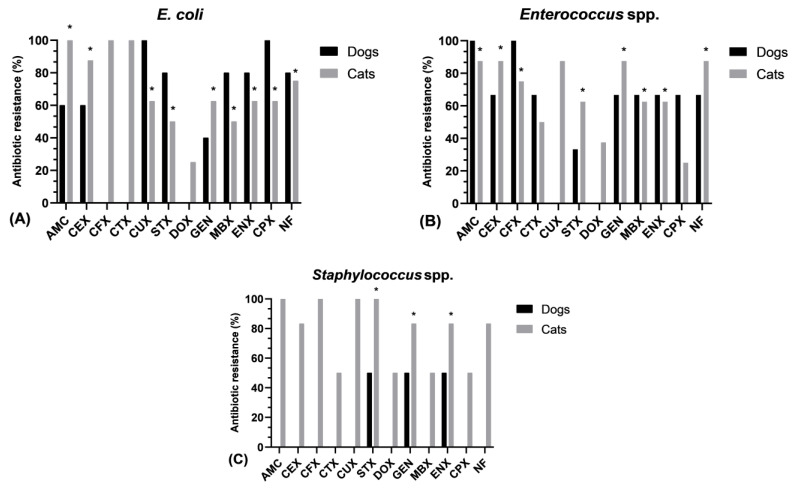
Antimicrobial resistance prevalence of common bacteria isolated from urinary tract infections in both species (dogs and cats); (**A**) Antimicrobial resistance of *Escherichia coli*, (**B**) Antimicrobial resistance of *Enterococcus* spp. (**C**) Antimicrobial resistance of *Staphylococcus* spp.; * Significant difference *p* < 0.05. AMC—Clavulanate amoxicillin, CEX—Cephalexin, CFX—Cefotaxime, CTX—Ceftriaxone, CUX—Cefuroxime, STX—Trimethoprim/sulfamethoxazole, DOX—Doxycycline, GEN—Gentamicin, MBX—Marbofloxacin, ENX—Enrofloxacin, CPX—Ciprofloxacin, NF—Nitrofurantoin.

The data analysis revealed a substantial variation in the prevalence of resistance of *Escherichia coli* isolates against clavulanate amoxicillin (*p* = 0.009), cephalexin (*p* = 0.016), marbofloxacin and trimethoprim/sulfamethoxazole (*p* = 0.045), nitrofurantoin (*p* = 0.022), ciprofloxacin and cefuroxime (*p* = 0.025), gentamicin (*p* = 0.041), and enrofloxacin (*p* = 0.032) between dogs and cats. In the case of doxycycline, cefotaxime, and ceftriaxone, only cat-specific *E. coli* isolates were resistant (Figure 1A). Additionally, a significant difference in resistance frequency between both species was also observed for *Enterococcus* spp. towards clavulanate amoxicillin and cefotaxime (*p* = 0.016); cephalexin, nitrofurantoin, and gentamicin (*p* = 0.015); marbofloxacin and enrofloxacin (*p* = 0.041); and trimethoprim/sulfamethoxazole (*p* = 0.037). The resistance against doxycycline and cefuroxime was observed only in feline *Enterococcus* bacteria (Figure 1B). In terms of *Staphylococcus* spp., the resistance frequency varied significantly between canines and felines against gentamicin, enrofloxacin (*p* = 0.037), and trimethoprim/sulfamethoxazole (*p* = 0.021). Similarly, feline staphylococci were resistant to the remaining antibiotics (Figure 1C).

### 2.4. Phytochemical Profile of Cornus mas L. and Sorbus aucuparia L. Fruit Extracts

The findings regarding the fruits’ phytochemical profiles were presented in prior studies performed in our lab [19,32] and are systematically revealed in Tables S1 and S2 as Supplementary Information.

### 2.5. Antimicrobial Activity of Cornus mas L. and Sorbus aucuparia L. Fruit Extracts on Resistant Clinical Isolates

#### 2.5.1. Selected Bacterial Isolates

To assess the antimicrobial activity of *Cornus mas* L. and *Sorbus aucuparia* L. fruit extracts, 13 clinical isolates were selected for testing (Table S3). Regrettably, *Leclercia adecarboxylata* was not analyzed due to loss of viability after preservation. Because *Escherichia* and *Proteus* were two of the most well-represented genera, two isolates from each were chosen for testing. In addition, no strains from the genus *Kocuria* were examined due to uncertainty regarding their pathogenicity and role in the development of urinary infections.

#### 2.5.2. Antimicrobial Activity by the Agar Well Diffusion Method

Both fruit extracts registered in vitro antimicrobial activity against all analyzed resistant bacteria. In the case of *Cornus mas* L. extract, the highest diameter of the inhibition zone was recorded towards *Ps. luteola* (16.70 ± 0.14 mm) for Gram-negative bacteria, being comparable to gentamicin’s control (*p* > 0.05). The lowest diameter was registered against *K. pneumoniae* (8.56 ± 0.04 mm) followed by *E. coli* 531 (8.75 ± 0.07 mm), with both strains being classified as multidrug resistant. Moreover, *Cornus mas* L. extract also showed promising antimicrobial activity against *P. mirabilis* isolates (15.00 ± 0.00 mm for sample no. 422 and 14.50 ± 0.21 mm for sample no. 582) and moderate activity towards *K. oxytoca* (12.35 ± 0.07 mm), *E. cloacae* (11.74 ± 0.07 mm), and *E. coli* 612 (10.73 ± 0.09 mm). However, all abovementioned diameters were significantly reduced in comparison to gentamicin control (*p* < 0.05). Remarkably, *Ac. baumannii* demonstrated resistance to gentamicin but was susceptible to *Cornus mas* L. extract, with an inhibitory zone diameter of 12.92 ± 0.10 mm (Figure S1).

*Sorbus aucuparia* L. extract displayed the highest level of effectiveness against *Ac. baumannii*, with an inhibition zone of 15.88 ± 0.02 mm. A comparable result was attained with *K. oxytoca* (14.31 ± 0.02 mm), followed by *E. cloacae* (13.19 ± 0.01 mm) and *E. coli* 612 (13.13 ± 0.09 mm). Interestingly, the lowest diameter of the inhibition zone was observed in the cases of *Ps. luteola* (10.60 ± 0.00 mm) and *K. pneumoniae* (10.93 ± 0.19 mm). Additionally, *Sorbus aucuparia* L. extract registered moderate activity towards *E. coli* 531 (11.81 ± 0.07 mm) and *P. mirabilis* strains (11.13 ± 0.19 mm and 11.12 ± 0.11 mm). Except *Ac. baumannii*, all inhibitory zones of *Sorbus aucuparia* L. extract were significantly reduced compared to gentamicin control (*p* < 0.05) (Figure S1).

Regarding Gram-positive bacteria, *E. faecalis*, *E. faecium*, and *S. pseudintermedius* showed resistance to the amoxicillin control, while *S. lentus* registered a small inhibition zone of 9.20 ± 0.007 mm. Both *Cornus mas* L. and *Sorbus aucuparia* L. extracts recorded substantial larger inhibitory zones against *S. lentus* compared to the control (14.56 ± 0.06 mm and 18.90 ± 0.141 mm, respectively; *p* < 0.05). Additionally, out of all Gram-positive bacteria, this was the greatest zone of inhibition registered by *Sorbus aucuparia* L. extract. The berry extract also demonstrated antimicrobial action against the remaining pathogens: *E. faecalis*—18.49 ± 0.007 mm, *S. pseudintermedius*—15.05 ± 0.07 mm, and *E. faecium*—13.13 ± 0.014 mm. Similar to *Sorbus aucuparia* L. extract, bacterial isolates were also sensitive to the *Cornus mas* L. extract: *S. pseudintermedius*—15.15 ± 0.06 mm, *E. faecalis*—14.79 ± 0.29 mm, and *E. faecium*—12.10 ± 0.14 mm. *Cornus mas* L. extract’s zone of inhibition obtained the largest diameter when applied to *S. pseudintermedius* (Figure S2).

**Figure 2:**
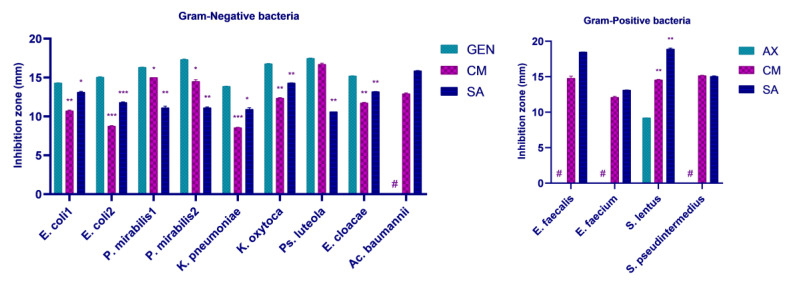
Antimicrobial activity of *Cornus mas* L. and *Sorbus aucuparia* L. extracts by disk diffusion method against resistant UTI bacteria. Significant differences vs. controls * *p* < 0.05, ** *p* < 0.01, *** *p* < 0.001; control of Gram-negative bacteria: GEN—gentamicin; control of Gram-positive bacteria: AX—amoxicillin; ^#^ isolate resistant to control (0.0 mm); CM—*Cornus mas* L.; SA—*Sorbus aucuparia* L.

All results are illustrated graphically in Figure 2.

#### 2.5.3. Antimicrobial Activity by the Broth Microdilution Method

The MIC and MBC values associated with each tested bacterial strain are shown in Table 3. As such, the lowest concentration of *Cornus mas* L. extract which showed the greatest inhibitory activity was 0.01 μg/μL. It was exhibited against Gram-positive *E. faecalis*. The following concentration, 0.02 μg/μL, was observed to be effective against Gram-negative *E. coli* 612 and *Ps. luteola*. The growth of Gram-negative *E. coli* 531, *Klebsiella* spp., and Gram-positive *S. lentus* was observed to be inhibited by the seventh concentration (0.04 μg/μL). Moreover, the initial recorded dosage that demonstrated both inhibitory and bactericidal activity was 0.08 μg/μL. It inhibited both *P. mirabilis* strains, *E. cloacae*, *Ac. baumannii*, and Gram-positive *E. faecium* and *S. pseudintermedius*. The bactericidal efficacy was manifested towards Gram-negative *P. mirabilis 582* and *Ps. luteola* and Gram-positive *E. faecalis* and *S. pseudintermedius*. The remaining pathogens were killed by the 0.16 μg/μL dosage. Furthermore, according to the MIC index value, *Cornus mas* L. extract manifested a bactericidal effect against nearly all analyzed resistant strains except for Gram-negative *E. coli* 612 and Gram-positive *E. faecalis*, on which it produced a bacteriostatic impact (Figure S3).

Regarding *Sorbus aucuparia* L. extract, the maximum inhibitory effect was observed against Gram-positive *E. faecalis* and Gram-negative *Klebsiella* spp. at a concentration of 0.01 μg/μL. Gram-negative *E. coli* 612 and Gram-positive *S. lentus* were suppressed by the 0.02 μg/μL concentration of extract, while all other bacteria were inhibited by the 0.05 μg/μL dosage. Additionally, the latter concentration was the first to show bactericidal activity, specifically towards Gram-negative *P. mirabilis* isolates. Moreover, three Gram-negative (*K. pneumoniae*, *K. oxytoca*, and *Ps. luteola*) and three Gram-positive (*E. faecalis*, *S. lentus*, and *S. pseudintermedius*) strains were shown to be susceptible to the bactericidal action of the 0.10 μg/μL dosage. The concentration of 0.19 μg/μL was able to kill Gram-negative *E. coli* 612, *E. cloacae*, and *Ac. baumannii*, together with Gram-positive *E. faecium*. Ultimately, the greatest extract concentration—0.39 μg/μL—was able to eradicate *E. coli* strain no. 531. Therefore, *Sorbus aucuparia* L. extract was shown to exhibit a bactericidal effect against Gram-negative *P. mirabilis* strains, *Ps. luteola, Ac. baumannii, E. cloacae* and Gram-positive *E. faecium*, *S. lentus*, and *S. pseudintermedius*, while the bacteriostatic action was manifested towards Gram-negative *Klebsiella* isolates and *E. coli* strains and Gram-positive *E. faecalis* (Figure S4).

## 3. Discussion

The major purpose of this study was to determine the in vitro antimicrobial activity of *Cornus mas* L. and *Sorbus aucuparia* L. fruit extracts against resistant bacterial isolates from UTIs in companion animals as well as to investigate the prevalence of urinary pathogens in dogs and cats. Initially, animal characteristics were analyzed as possible indicators of UTI occurrence in pets. As such, the distribution of species and sex within the entire group was fairly equal, suggesting their minimal impact on the emergence of UTIs. However, the male population outnumbered the female group. Ataya et al. [37] obtained similar results, although the proportion was opposite for females compared to males. Additionally, comparable findings about the species distribution were observed in a prior work [38]. Other studies have indicated the importance of neuter status on the incidence of UTI, with sterilized females being more inclined to experience urinary infections [2,39]. Moreover, mature dogs and young cats appeared to be particularly susceptible. According to the data in the literature, elderly animals appear to be more impacted, which is anticipated considering the multitude of risk factors encountered at advanced ages [40,41]. The increased prevalence of young cats in our study could be attributed to the frequent diagnosis of urethral obstructions in these animals, which necessitates urine catheterization and thus provides a potential entrance point for bacterial infections [42]. Regarding antibiotic usage, animals with UTIs who had not previously used antibiotics were more commonly encountered. Some authors have indicated that ongoing UTIs are frequently linked to persistent antibiotic treatment throughout time [2,43]. Furthermore, purebred dogs and domestic cats tended to be more vulnerable. Regarding felines, our outcomes are in agreement with a previously mentioned study [38]. However, those authors highlighted the more frequent incidence in common breed dogs.

Regarding the uropathogens, the diversity of bacterial genera was higher among dogs, while antimicrobial resistance was more significant among feline clinical isolates. Gram-negative isolates predominated over Gram-positive strains, in accordance with earlier studies [2,11]. *Escherichia coli* was the most frequent Gram-negative bacteria, with 13 strains, ranking first in cats and second in dogs. It is considered the main contributory factor of UTIs [44,45,46,47] as well as a commensal bacterium in the intestinal microflora, spreading rapidly through feces [48,49]. Both feline and canine *E. coli* isolates were completely resistant to specific cephalosporins (cefotaxime, ceftriaxone, and cefuroxime). Cephalosporin resistance has been previously reported [45,50], and ongoing surveillance is required to increase the effectiveness of these medicines. Moreover, *Proteus mirabilis* was the second most prevalent Gram-negative bacterium (seven strains), found solely in dogs, as described earlier by other researchers [11,51]. Given its inherent resistance to several antibiotics, its evolved resistance to fluoroquinolones, and its possible zoonotic involvement in human UTIs, this bacterium is of significant importance [2,52,53]. In the current research, full resistance was registered towards marbofloxacin fluoroquinolone, among other antibiotics. Furthermore, another dog-specific Gram-negative bacterium identified in urine samples was *Klebsiella pneumoniae*. Importantly, it registered the highest level of antimicrobial resistance, showing no susceptibility to 10 of the 12 evaluated antibiotics. Over the years, *K. pneumoniae* has turned into a major public health concern in both human and veterinary medicine due to its rising drug-resistant characteristics [5]. Other Gram-negative resistant bacteria included *Pseudomonas luteola*, *Acinetobacter baumannii*, *Enterobacter cloacae*, and *Klebsiella oxytoca*. Similarly, some authors have identified a few of these strains [2,7,52]. 

Additionally, a rarely documented Gram-negative bacteria in UTI cases discovered was *Leclercia adecarboxylata*. It is an agile bacillus that is extensively distributed in nature and a member of the *Enterobacteriaceae* family. *L. adecarboxylata* is a typical component of the intestinal microbiota in humans and animals, and when pathogenic, can induce a variety of diseases, including septicemia, peritonitis, and UTIs [54]. A prior study also confirmed the presence of this pathogen in a canine UTI patient [52].

*Enterococcus* spp. was the most common pathogen among Gram-positive bacteria, second only to *E. coli*, and was found primarily in cats. The results of prior studies reinforce this finding [7,11]. Of *Enterococcus* spp., *E. faecalis* and *E. faecium* were the two pathogens encountered. *Enterococcus* isolates registered 100% resistance to clavulanate amoxicillin and cefotaxime. Other studies reported resistance to doxycycline and fluoroquinolones rather than beta-lactams [2,55]. The variations in resilience patterns may be due to regional and time-dependent changes in antibiotic consumption habits [47]. Therefore, due to their inbuilt opposition, gained aversion to fluoroquinolones and tetracyclines, and selective ability to induce complicated nosocomial human infections, there may exist an alarming reduction in the odds of managing Enterococci infections [5]. 

Furthermore, *Staphylococcus* spp. was the next predominant Gram-positive bacterium, appearing in both species but more frequently in cats. Specifically, *S. pseudintermedius* was recovered from both species; *S. aureus* was a dog-specific isolate, while *S. lentus*, *S. equorum*, *S. haemolyticus*, and *S. sciuri* were detected only in felines. It seems that the majority of *S. aureus* strains found in pet species were linked to distinct human strains, while *S. pseudintermedius* and *S. sciuri* have recently been thought to be involved in human zoonotic infections [56,57,58]. Additionally, the risk factors for *S. haemolyticus* in cats are similar to that seen in *S. aureus*-infected animals [59]. In the present study, feline *Staphylococcus* spp. was entirely resistant to several beta-lactams (clavulanate amoxicillin, cefotaxime, and cefuroxime) and trimethoprim/sulfamethoxazole, while only half of the canine strains were resistant to trimethoprim/sulfamethoxazole, gentamicin, and enrofloxacin. Several authors have mentioned susceptibility to other beta-lactams (mainly oxacillin), fluoroquinolones, and aminoglycosides [2,52,60]. As previously stated, geography and time-related drug uses may alter these bacteria’s resistance profiles. 

Moreover, two unusual Gram-positive *Kocuria* strains (*K. rosea* and *K. rhizophila*) were isolated from dog urine samples. The *Kocuria* genus, belonging to the *Micrococcaceae* family, are commensals of human skin and mouth microbiota as well as potential opportunistic pathogens; however, human infections have been uncommonly reported [61,62]. A recent study demonstrated that *Kocuria* spp. can be mistaken for coagulase-negative staphylococci in human UTIs [63]. As for animals, a study identified *Kocuria* spp. isolates in the atmosphere of a military dog facility [64]. Therefore, to the best of our knowledge, this is the first study to discover *Kocuria* spp. pathogens in companion animal UTIs.

In terms of fruit extracts, *Cornus mas* L. and *Sorbus aucuparia* L. demonstrated antimicrobial efficacy towards all 13 resistant uropathogens. It could be observed that *Ps. luteola* was particularly sensitive to *Cornus mas* L., while *E. faecalis* was especially susceptible to *Sorbus aucuparia* L., the latter having a greater significance in the prevalence of UTIs. Furthermore, both extracts demonstrated superior efficacy against Gram-positive bacteria when compared to amoxicillin control; nevertheless, they were unable to outperform gentamicin against Gram-negative bacteria. Overall, *Cornus mas* L. extract was more efficient against Gram-negative bacteria, whilst the effectiveness of *Sorbus aucuparia* L. extract was higher towards Gram-positive organisms. Additionally, *Cornus mas* L. extract was able to kill more bacteria than *Sorbus aucuparia* L. extract.

Since both fruit extracts have been demonstrated to contain great amounts of polyphenols, among other compounds, their antimicrobial activity could be attributed to these phytochemicals. Polyphenols have been used as adjuvants to antibiotics for enhancing their efficiency, reducing their dose, and thus lowering unfavorable responses. A possible underlying process involves the destabilization of the bacterial cell structure through notable alterations in the membrane’s lipid bilayer, increasing in permeability and interaction with resistant proteins, thereby amplifying an antibiotic’s effectiveness against bacteria that were previously resistant to its activity [65]. Previous research demonstrated that using multiple antibiotics in combination with gallic acid, the major phenolic compound identified in *Cornus mas* L. fruits in our investigation, may produce a synergistic effect [34]. Additionally, proanthocyanidins, which are found in high quantities in *Sorbus aucuparia* L. fruits, enhanced the antifungal activity of bifonazole and ketoconazole [66]. Our findings suggest that *Cornus mas* L. and *Sorbus aucuparia* L. fruit extracts could be utilized in conjunction with at least gentamicin and amoxicillin for the management of resistant Gram-positive and Gram-negative clinical isolates. Moreover, the specific use of these fruit extracts together with amoxicillin, which demonstrated low activity, may help restore its antimicrobial efficiency. In essence, the main potential implication of these fruit extracts could be a slowdown of resistance growth since the antimicrobial action might be accomplished with lower antibiotic dosages. Additionally, the inhibition of resistant bacteria with plant extracts, such as *Cornus mas* L. and *Sorbus aucuparia* L., may be vital for achieving a successful and durable therapy.

Recent advancements in herbal medicine present novel opportunities for *Cornus mas* L. usage in the management of UTIs. Thus, few studies have previously been reported. In a rat experimental model, *Cornus mas* L. was demonstrated to be a viable replacement for nitrofurantoin in UTI treatment, registering similar effects [67]. The oral administration of *Cornus mas* L. for seven days decreased inflammation in rats with experimentally induced interstitial cystitis [68]. Additionally, a 6-month oral therapy experiment showed that *Cornus mas* L. reduced dysuria and frequent urination in women with UTIs [69]. Nevertheless, additional research is required to strengthen the hypothesis of the antimicrobial properties of *Cornus mas* L. fruits.

To the best of our knowledge, there has only been one previously published study on *Sorbus aucuparia* L.‘s antibacterial properties in the management of UTIs, which unfortunately yielded poor results [70].

Research into therapeutic plants’ impacts on resistant UTI bacteria in companion animals is currently limited. A study conducted by Ebani et al. [71] investigated the antimicrobial potential of several essential oils against *E. coli* and *Enterococcus* strains of UTIs in dogs and cats. Essential oils of *I. verum* L. and *S. sclarea* L. registered moderate activity against some *E. coli* strains, while no effect was observed towards *Enterococcus* spp. *O. vulgare* L. and *T. vulgaris* L. oils recorded promising effects against both *E. coli* and *Enterococcus* spp. In comparison, *Cornus mas* L. and *Sorbus aucuparia* L. extracts reported a greater antimicrobial activity against *Enterococcus* spp. Moreover, recent studies have focused on the in vivo impact of cranberry extract, which has been proven to possess antibacterial qualities that might assist in preventing or minimizing the onset of UTIs in canine patients [72,73]. Additionally, *Boerhaavia diffusa* L., another intriguing herbal medication, showed evidence of in vivo antimicrobial activity in treating bacterial cystitis in senior dogs, the urine culture being negative on the fifteenth day of treatment [74]. In light of this, more research into medicinal plants with potential antimicrobial capabilities is essential to reduce antibiotic resistance of bacteria associated with companion animal diseases.

As a whole, it should be highlighted that this is the first study to demonstrate the antimicrobial activity of *Cornus mas* L. and *Sorbus aucuparia* L. fruit extracts against resistant clinical isolates retrieved from companion animals UTIs. This paper might inspire the more frequent use of antibiotics in conjunction with natural compounds, hence minimizing antibiotic resistance.

## 4. Materials and Methods

### 4.1. Reagents and Chemicals

The standard and special culture media were acquired from BioMaxima S.A, Lublin, Poland, and Bio-Rad Laboratories Inc., Hercules, CA, USA, respectively. Mueller-Hinton agar and broth were supplied by Merck, Darmstadt, Germany, while Liofilchem, Teramo, Italy provided the antibiotic disks. The remaining substances were bought from Sigma Aldrich, Darmstadt, Germany.

### 4.2. Plant Material

Fruits of *Cornus mas* L. and *Sorbus aucuparia* L. were gathered from trees situated on the slopes in Cluj County, Romania’s Mărişel settlement (46°40′03.7′′ N 23°06′35.6′′ E). The berry collection occurred between August and September of 2021. In an attempt to identify the plant species, branches with leaves and flowers were also harvested in March–April 2021. After the plants were recognized, the fruit flesh was manually separated from the seeds and further distributed in specimen bags containing 10 g of flesh each. The bags were preserved at −18 °C until further examination.

### 4.3. Fruit Extract Preparation

Initially, each fruit category was unfrozen and air dried using a fruit dehydrator (model TEESA TSA3031, Lechpol Electronics Leszek Sp. K., Garwolin, Poland) fixed at 45 °C for one week. After that, the desiccated fruits were finely powdered. Hydro-alcoholic extracts were prepared from both fruit powders per a previous study [19], with slight changes. In brief, 10 g of each fruit powder together with 100 mL ethanol 96% (Sigma Aldrich, Darmstadt, Germany) were mixed for 2 h (with a magnetic stirrer—VELP Scientifica, Usmate Velate, Italy), filtered, re-extracted, and evaporated at 45 °C (with an Eppendorf evaporator—Hamburg, Germany) until the ethanol was completely dispersed. Finally, the resulting concentrated fruit extracts were reconstituted with distilled water and further employed for subsequent analysis. 

The quantitative assessments comprised total phenolic, flavonoid, and carotenoid concentrations, while the qualitative analysis included phenolic compound identification utilizing HPLC-DAD-ESI-MS. All measurements were described and presented in detail in previous investigations conducted in our lab [19,32].

### 4.4. Biological Material

The biological samples included in the study were represented by urine samples provided to the Department of Microbiology, Immunology, and Epidemiology of the Faculty of Veterinary Medicine, Cluj-Napoca, Romania, that were sent in as part of routine microbiological diagnoses. All urine specimens were collected from patients with naturally occurring diseases. The companion animal samples (dogs and cats) incorporated into the study were gathered and processed between June 2022 and July 2023. Each urine sample was accompanied by a dispatch note containing data about the owner, the animal (species, breed, age, and gender), and the technique used for sample collection (spontaneous micturition, catheterization, or cystocentesis). It was also specified, where applicable, whether the UTI was recurrent and whether antibiotic therapies had been administered. 

Throughout the collection and processing period, urine samples were obtained from 83 animals, of which 47 were negative and 36 were positive. Subjects who tested positive for infection also included patients from which several bacterial strains were isolated as well as patients with recurrent UTIs from which repeated samples were processed. Statistical analyses and results interpretation focused solely on the positive-tested samples.

### 4.5. Preliminary Identification and Antimicrobial Susceptibility Testing

All urine specimens underwent processing on the day they were presented to the lab. Inoculations were performed on common and special culture media for the urinary pathogens detection: Columbia agar with 10% sheep blood (BioMaxima S.A, Lublin, Poland), special UriSelect medium (Bio-Rad Laboratories Inc., Hercules, CA, USA) and nutrient agar (Merck, Darmstadt, Germany), respectively, for colony quantification. After incubation at 37 °C for 24 h, the colonies’ cultural characteristics were evaluated following the manufacturer’s recommendations; morphological assessment was carried out using Gram staining, and colony counting was assessed using a colony counter (model CC-1, Boeco, Hamburg, Germany).

The diagnosis of bacterial UTI was made by comparing the total number of bacterial colonies found through quantitative analysis with the anamnesis and clinical symptoms linked to the infection. Thus, depending on the urine sample collection method, the following criteria were used: total number of germs (TNG) ≥ 100,000 CFU/mL for spontaneous micturition, ≥10,000 CFU/mL for catheterization in males, and ≥100,000 CFU/mL for catheterization in females. Any bacterial growth was considered in cases when the sample was obtained via cystocentesis [75].

The antibiotic susceptibility testing was carried out using the disk diffusion method in compliance with the EUCAST guideline [76]. Briefly, a bacterial suspension was obtained in sterile saline (NaCl 0.9%, Sigma Aldrich, Darmstadt, Germany), having a 0.5 density on the McFarland scale, and inoculated on Mueller-Hinton (MH) agar plates (Merck, Darmstadt, Germany) via the three-section approach. The following commercial antibiotic disks (Liofilchem, Teramo, Italy) were radially placed onto the plates and used for testing: beta-lactam antibiotics (clavulanate amoxicillin 10–20 μg; cephalosporins: cephalexin 30 μg, cefotaxime 5 μg, ceftriaxone 30 μg, cefuroxime 30 μg), aminoglycosides (gentamicin 10 μg), tetracyclines (doxycycline 30 μg), nitrofurantoin 100 μg, fluoroquinolones (enrofloxacin 5 μg, marbofloxacin 5 μg, ciprofloxacin 5 μg), and trimethoprim/sulfamethoxazole 1.23–23.75 μg [77]. 

Following sample preparation, incubation at 35 ± 1 °C for 16–20 h was performed, after which the diameters of the inhibition zones for each antibiotic were measured. Based on EUCAST breakpoints [78], the bacteria were categorized as susceptible (S), resistant (R), or intermediate (I). 

Following diagnosis, isolated strains from each sample were kept in tubes containing 60% glycerine broth at −20 °C until additional processing.

### 4.6. Bacterial Species Identification

For identification, bacterial isolates were replated on solid culture media (10% sheep blood Columbia agar). Using the Vitek^®^ 2 Compact device (BioMerieux, Marcy l’Etoile, France), species identification was conducted in precise accordance with the manufacturer’s instructions. The Vitek^®^ system evaluates 64 distinct biochemical properties to identify microorganisms by using particular colorimetric cards. Hence, GP cards were utilized for Gram-positive bacteria and GN cards for Gram-negative bacteria [79].

### 4.7. Bacterial Isolates Selection for Fruit Extracts Antimicrobial Activity Assay

An attempt was made to examine at least one strain from each genus whose species had been discovered. Thus, bacterial strains that were deemed to be the most aggressive and that fulfilled one or more of the following requirements were selected for antimicrobial activity testing: bacteria isolated from animals with recurrent urinary infections and with previous antibiotic treatments, strains that presented hemolytic activity when cultured on blood agar, and resistance to the abovementioned antibiotics. 

### 4.8. Antimicrobial Activity of Investigated Fruit Extracts

#### 4.8.1. Agar Well Diffusion Method

The disk diffusion method, based on the EUCAST standards [76], was carried out according to the protocol described for the susceptibility test. After inoculating the MH agar plates with bacterial suspension, four 6 mm diameter wells were formed, and 60 μL of each solution—*Cornus mas* L. extract, *Sorbus aucuparia* L. extract, ethanol/distilled water (*v*/*v*), and dimethyl sulfoxide (DMSO) as a negative control—were added. The extracts’ concentrations were established using the total polyphenolic assay results, being 5.12 μg GAE/μL for *Cornus mas* L. extract and 6.23 μg GAE/μL for *Sorbus aucuparia* L. extract. Additionally, the proper antibiotic disk was placed in the center of each plate; amoxicillin (AX 10 μg, Liofilchem, Teramo, Italy) was used as a control for Gram-positive strains, whereas gentamicin (GEN 30 μg, Liofilchem, Teramo, Italy) was used as a control for Gram-negative bacteria. Following incubation at 37 °C for 24 h, the diameters of the inhibition zones were measured and expressed in mm. All measurements were performed in accordance with Vlase et al. [80], with minor modifications, and were run in duplicate.

#### 4.8.2. Determination of Minimum Inhibitory Concentration (MIC) Index

This assessment was carried out in line with Widelski et al. [81], with few modifications, and included the measurement of the minimal inhibitory concentration (MIC) and the minimal bactericidal concentration (MBC). Regarding MIC analysis, sterile 96-microwell plates (Millicell^®^, Sigma Aldrich, Darmstadt, Germany) were used to perform two-fold serial dilutions of the tested extracts in MH broth. The dilutions corresponded to extract concentrations ranging from 2.56 to 0.005 μg GAE/μL *Cornus mas* L. extract and from 3.11 to 0.006 μg GAE/μL *Sorbus aucuparia* L. extract; these values were computed using the total phenolic content. Furthermore, 20 μL of bacterial suspension (10^6^ CFU/mL) from each strain was added to all wells. The ethanol/distilled water mixture (*v*/*v*) served as a negative control, while MH broth together with the bacterial suspension served as a positive control. The plates were then incubated at 37 °C for 24 h. The absence of turbidity in the well indicated the inhibitory effect, and the MIC value was established by recording the lowest concentration able to inhibit visible microbial growth.

For every strain and extract, the minimum bactericidal concentration (MBC) was also assessed. As such, 10 μL of each well—where no bacterial growth was observed—was seeded into MH agar plates. The plates were then incubated for 24 h at 37 °C.

The MIC index was computed based on the MBC/MIC ratio to ascertain if each extract had a bacteriostatic (MBC/MIC > 4) or bactericidal (MBC/MIC ≤ 4) effect on the tested bacterial strains.

### 4.9. Statistical Analysis

Data analysis was performed using the Graph Pad Prism 8 (San Diego, CA, USA) software and Microsoft Office Excel 2016. The risk factors, pathogen prevalence, and susceptibility to antimicrobials were analyzed utilizing the chi-squared independence test. The antimicrobial activity of fruit extracts was examined employing one-way ANOVA and *t*-test functions; *p* < 0.05 was used as the significance threshold.

## 5. Conclusions

*Cornus mas* L. and *Sorbus aucuparia* L. fruit extracts appear to be viable natural remedies for treating resistant bacteria seen in dogs and cats with UTIs. Both fruit extracts showed antimicrobial efficacy against all tested resistant strains, with *Cornus mas* L. expressing better activity against Gram-negatives, and *Sorbus aucuparia* L. having a significant effect on Gram-positives. Their antimicrobial efficiency could be determined by their enhanced polyphenolic content, which suggests their role as adjuvants to commercial antibiotics, particularly amoxicillin, boosting their antimicrobial activity. The implementation of these fruit extracts in the treatment of companion animal UTIs may lead to a reduction in human antibiotic usage in veterinary medicine as well as the use of low-dose antibiotics, thus minimizing the development of antimicrobial resistance. Additionally, a novel aspect of this paper was the detection of pathogenic bacterium from the *Kocuria* genus in canine patients suffering from UTIs. In its entirety, this article provides originality and demonstrates for the first time the antimicrobial effect of *Cornus mas* L. and *Sorbus aucuparia* L. fruit extracts against resistant clinical strains isolated from pets diagnosed with UTIs.

## Figures and Tables

**Table 1:** Characteristics of investigated dogs and cats.

	Dogs %			Cats %			Total
Gender	Male	Female		Male	Female		36
	55 (*n* = 11)	45 (*n* = 9)		56.25 (*n* = 9)	43.75 (*n* = 7)		
Age	Young	Adult	Geriatric	Young	Adult	Geriatric	31 *
	37.5 (*n* = 6)	50 (*n* = 8)	12.5 (*n* = 2)	53.34 (*n* = 8)	33.33 (*n* = 5)	13.33 (*n* = 2)	
Breed	Yes	No		Yes	No		36
	65 (*n* = 13)	35 (*n* = 7)		12.5 (*n* = 2)	87.5 (*n* = 14)		
Antibiotic usage	Yes	No		Yes	No		36
	30 (*n* = 6)	70 (*n* = 14)		25 (*n* = 4)	75 (*n* = 12)		

* 31 animals analyzed based on age; 5 animals with unknown age. Young = 0–3 years; adult = 3–9 years; geriatric ≥ 9 years.

**Table 2:** Bacterial strains from analyzed urine samples of companion animals.

Bacterial Isolates	% in Dogs	% in Cats	Total	*p* Value
* Escherichia coli * *	20 (*n* = 5)	33.33 (*n* = 8)	13	*** p * = 0.0088 **
* Enterococcus faecalis * *	8 (*n* = 2)	20.83 (*n* = 5)	7	*** p * = 0.0418 **
* Proteus mirabilis *	28 (*n* = 7)	0	7	N /A
* Enterococcus faecium *	4 (*n* = 1)	12.5 (*n* = 3)	4	* p * = 0.1138
* Klebsiella pneumoniae * *	8 (*n* = 2)	0	2	N /A
* Acinetobacter baumannii *	4 (*n* = 1)	4.17 (*n* = 1)	2	* p * = 0.3173
* Enterobacter cloacae *	4 (*n* = 1)	0	1	N/A
* Klebsiella oxytoca *	4 (*n* = 1)	0	1	N/A
* Staphylococcus aureus *	4 (*n* = 1)	0	1	N/A
* Staphylococcus pseudintermedius *	4 (*n* = 1)	4.17 (n = 1)	2	* p * = 0.3173
* Staphylococcus lentus *	0	8.33 (*n* = 2)	2	N/A
* Staphylococcus equorum *	0	4.17 (*n* = 1)	1	N/A
* Staphylococcus sciuri *	0	4.17 (*n* = 1)	1	N/A
* Staphylococcus haemolyticus *	0	4.17 (*n* = 1)	1	N/A
* Pseudomonas luteola *	4 (*n* = 1)	0	1	N/A
* Kocuria rosea *	4 (*n* = 1)	0	1	N/A
* Kocuria rhizophila *	4 (*n* = 1)	0	1	N/A
* Leclercia adecarboxylata *	0	4.17 (*n* = 1)	1	N/A
Total	* n * = 25	* n * = 24	49	N/A

N/A = not applicable. Bold—significant difference *p* < 0.05; * one isolate identified in repeatedly processed urine samples.

**Table 3:** Antimicrobial activity of *Cornus mas* L. and *Sorbus aucuparia* L. extracts by broth microdilution method against resistant UTI isolates.

MIC Index MIC (μg GAE/μL)/MBC (μg GAE/μL)		
Bacterial Isolates	*Cornus mas* L.	*Sorbus aucuparia* L.
*E. coli* 1	**8**	**8**
	0.16/0.02	0.19/0.02
*E. coli* 2	**4**	**8**
	0.16/0.04	0.39/0.05
*P. mirabilis* 1	**2**	**1**
	0.16/0.08	0.05/0.05
*P. mirabilis* 2	**1**	**1**
	0.08/0.08	0.05/0.05
*K. pneumoniae*	**4**	**8**
	0.16/0.04	0.10/0.01
*K. oxytoca*	**4**	**8**
	0.16/0.04	0.10/0.01
*Ps. luteola*	**4**	**2**
	0.08/0.02	0.10/0.05
*E. cloacae*	**2**	**4**
	0.16/0.08	0.19/0.05
*Ac. baumannii*	**2**	**4**
	0.16/0.08	0.19/0.05
*E. faecalis*	**8**	**8**
	0.08/0.01	0.10/0.01
*E. faecium*	**2**	**4**
	0.16/0.08	0.19/0.05
*S. lentus*	**4**	**4**
	0.16/0.04	0.10/0.02
*S. pseudintermedius*	**1**	**2**
	0.08/0.08	0.10/0.05

Bold—MIC index value; 1 ≤ 4 = bactericidal effect, >4 = bacteriostatic effect.

## Data Availability

The original contributions presented in the study are included in the article/Supplementary Material, further inquiries can be directed to the corresponding author.

## Supplementary Materials

The following supporting information can be downloaded at: https://www.mdpi.com/article/10.3390/ph17060814/s1, Table S1. Total polyphenols, flavonoids, and carotenoids of *Cornus mas* L. and *Sorbus aucuparia* L. fruit extracts. Table S2. HPLC-DAD-ESI-MS identification of phenolic compounds of *Cornus mas* L. and *Sorbus aucuparia* L. fruit extracts. Table S3: Selection criteria for clinical isolates. Figure S1: Antimicrobial activity of *Cornus mas* L. (CM) and *Sorbus aucuparia* L. (SA) fruit extracts against resistant Gram-negative UTI bacteria in companion animals by agar well diffusion method; negative controls—mix of ethanol/distilled water (*v*/*v*) and DMSO; positive control—gentamicin (center). Figure S2: Antimicrobial activity of *Cornus mas* L. (CM) and *Sorbus aucuparia* L. (SA) fruit extracts against resistant Gram-positive UTI bacteria in companion animals by agar well diffusion method; negative controls—mix of ethanol/distilled water (*v*/*v*) and DMSO; positive control—amoxicillin (center). Figure S3: Antimicrobial activity of *Cornus mas* L. fruit extract against resistant UTI bacteria in companion animals by determining the minimum inhibitory concentration (MIC); NC—Negative control (mix of ethanol/distilled water *v*/*v*); PC—positive control (MH agar with bacterial suspension); 1—*E. coli* 612, 2—*E. coli* 531, 3—*P. mirabilis* 422, 4—*P. mirabilis* 582, 5—*K. pneumoniae*, 6—*K. oxytoca*, 7—*Ps. luteola*, 8—*E. faecalis*, 9—*E. faecium*, 10—*E. cloacae*, 11—*Ac. Baumannii*, 12—*S. lentus*, 13—*S. pseudintermedius*. Figure S4: Antimicrobial activity of *Sorbus aucuparia* L. fruit extract against resistant UTI bacteria in companion animals by determining the minimum inhibitory concentration (MIC); NC—Negative control (mix of ethanol/distilled water *v*/*v*); PC—positive control (MH agar with bacterial suspension); 1—*E. coli* 612, 2—*E. coli* 531, 3—*P. mirabilis* 422, 4—*P. mirabilis* 582, 5—*K. pneumoniae*, 6—*K. oxytoca*, 7—*Ps. luteola*, 8—*E. faecalis*, 9—*E. faecium*, 10—*E. cloacae*, 11—*Ac. Baumannii*, 12—*S. lentus*, 13—*S. pseudintermedius*.
