# Supplementary material for: The Antimicrobial Effect of Cornus mas L. and Sorbus aucuparia L. Fruit Extracts against Resistant Uropathogens in Correlation with the Prevalence of Urinary Tract Infections in Companion Animals
Source: Pharmaceuticals (Basel). 2024 Jun 20;17(6):814. doi: 10.3390/ph17060814 (PMC11206431; doi:10.3390/ph17060814)
Supplement: Supplementary file 1 [file pharmaceuticals-17-00814-s001.zip › pharmaceuticals-3043047-supplementary.pdf]

Table S1. Total polyphenols, flavonoids, and carotenoids of *Cornus mas* L. and *Sorbus aucuparia* L. fruit extracts [19,32]

Tested Extracts	Total Polyphenols (mg GAE/mL)	Total Flavonoids (µg QE/mL)	Total Carotenoids (µg/g)
Cornus mas L.	0.872 ± 0.0035	139.14 ± 2.10	3.8 ± 0.0002
Sorbus aucuparia L.	1.39 ± 0.046	537.58 ± 3.255	95.68 ± 0.297

Table S2. HPLC-DAD-ESI-MS identification of phenolic compounds of *Cornus mas* L. and *Sorbus aucuparia* L. fruit extracts [19,32]

Subclass	*Cornus mas* L.		*Sorbus aucuparia* L.	
	Compound	Concentration (µg/mL)	Compound	Concentration (µg/mL)
Phenolic acids	**Gallic acid glucoside**	**248.516**	Gallic acid-glucoside	61.799
	Chlorogenic acid	23.189	**Chlorogenic acid**	**704.792**
	Caffeic acid	57.150	**Neochlorogenic acid**	**376.610**
			Cryptochlorogenic acid	91.199
			Ferulic acid	45.018
Anthocyanins	Cy3-O-(coumaroyl-glucoside)	6.439	Cy 3-O-(caffeoyl-glucoside)	3.212
	Cy 3-O-galactoside<br>Cy 3-O-robinobioside	89.484	Cy 3-O-glucoside	19.237
	Pg 3-O-galactoside<br>Pg 3-O-robinobioside	42.826		
Flavonols	Rutin	29.635	Rutin	12.198
	K 3-O-galactoside	11.023	Q 3,4′-O -diglucoside	70.310
	**Procyanidin dimer**	**195.826**	Q 3-O-glucoside	24.139
Iridoids	Loganin	111.478	-	
	Sweroside	31.602		

Bold – major components of both fruit extracts.

Table S3. Selection criteria for clinical isolates

Isolate code	Clinical isolate	Sample code	Selection criteria
1	*Escherichia coli*	612/2022	haemolytic strain; resistant to clavulanate amoxicillin, cephalosporins, aminoglycosides, nitrofurantoin
2	*Escherichia coli*	531/2023	MDR strain; resistant to clavulanate amoxicillin, cephalosporins, fluoroquinolones, nitrofurantoin
3	*Proteus mirabilis*	422/2022	rUTI, treated with antibiotic; resistant to clavulanate amoxicillin, cephalosporins, fluoroquinolones, nitrofurantoin
4	*Proteus mirabilis*	582/2022	resistant to clavulanate amoxicillin, cephalosporins, fluoroquinolones, aminoglycosides, nitrofurantoin
5	*Klebsiella pneumoniae*	174/2023	rUTI, treated with antibiotic; MDR strain; resistant to clavulanate amoxicillin, cephalosporins, fluoroquinolones, nitrofurantoin
6	*Klebsiella oxytoca*	190/2023	rUTI, treated with antibiotic; resistant to clavulanate amoxicillin, cephalosporins, aminoglycosides, nitrofurantoin
7	*Pseudomonas luteola*	95/2023	resistant to nitrofurantoin
8	*Enterococcus faecalis*	60/2023	haemolytic strain; resistant to clavulanate amoxicillin, cephalosporins, nitrofurantoin, fluoroquinolones
9	*Enterococcus faecium*	226/2023	haemolytic strain; resistant to clavulanate amoxicillin, cephalosporins, nitrofurantoin, fluoroquinolones
10	*Enterobacter cloacae*	315/2023	resistant to clavulanate amoxicillin, cephalosporins, nitrofurantoin, fluoroquinolones, aminoglycosides
11	*Acinetobacter baumannii*	233/2023	rUTI, treated with antibiotic; resistant to clavulanate amoxicillin, cephalosporins, aminoglycosides, nitrofurantoin, fluoroquinolones
12	*Staphylococcus lentus*	707/2022	resistant to clavulanate amoxicillin, cephalosporins, nitrofurantoin, aminoglycosides
13	*Staphylococcus pseudintermedius*	103/2023	haemolytic strain; resistant to clavulanate amoxicillin, cephalosporins, aminoglycosides, fluoroquinolones

MDR = multidrug resistant bacteria; rUTI = recurrent urinary tract infection.

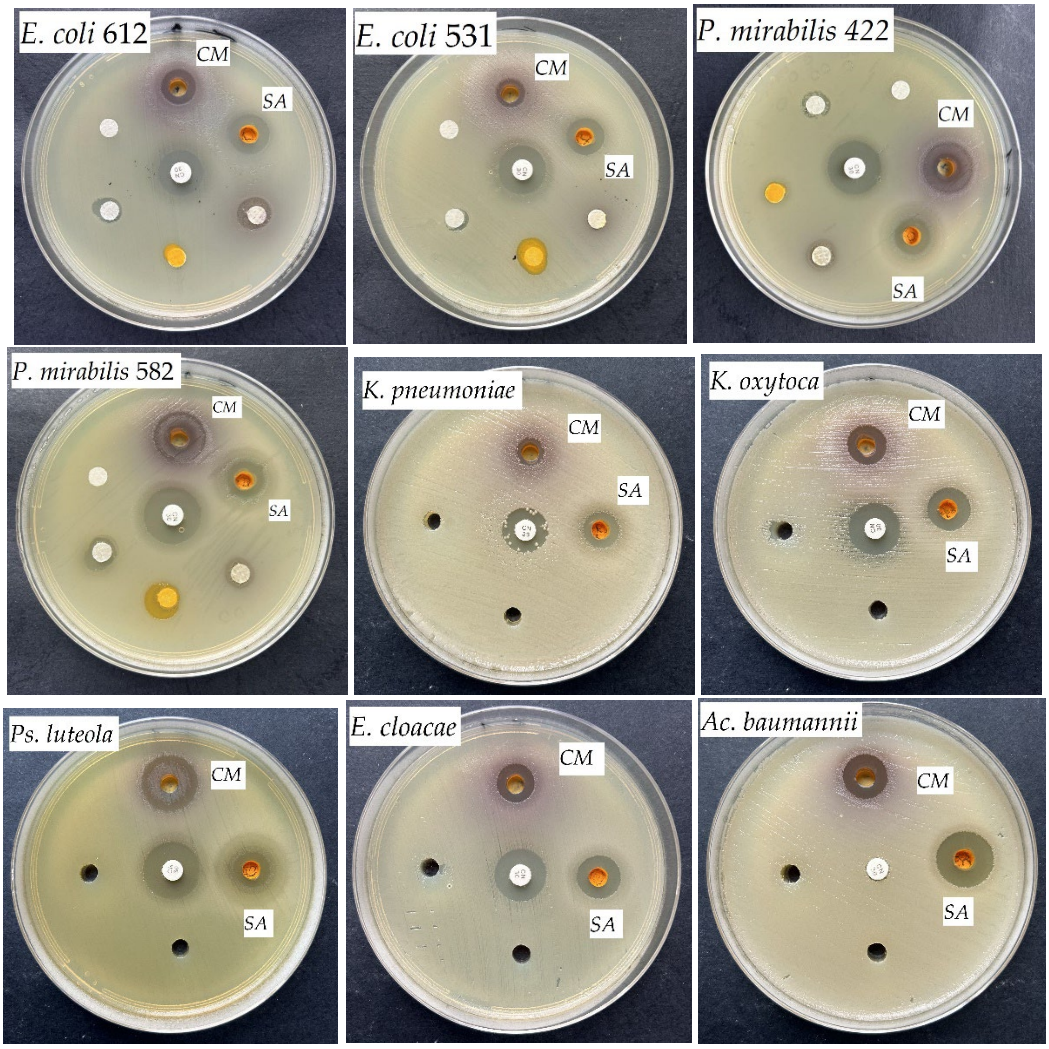

Figure S1. Antimicrobial activity of *Cornus mas* L. (CM) and *Sorbus aucuparia* L. (SA) fruit extracts against resistant Gram-negative UTI bacteria in companion animals by agar-well diffusion method; negative controls—mix of ethanol/distilled water (*v*/*v*) and DMSO; positive control—gentamicin (center).

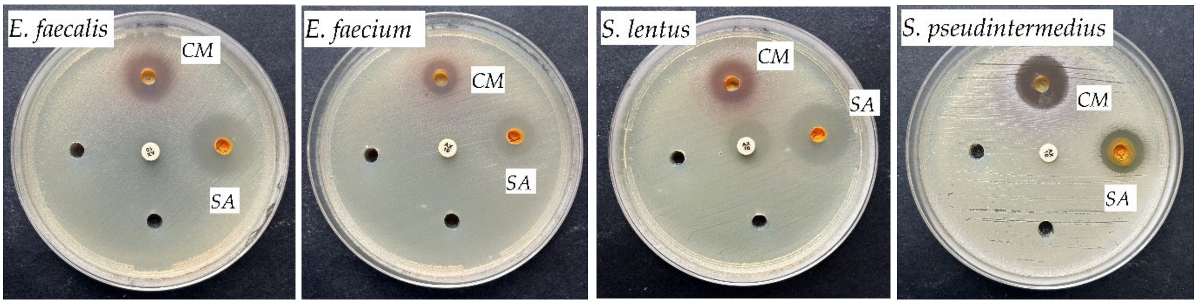

Figure S2. Antimicrobial activity of *Cornus mas* L. (CM) and *Sorbus aucuparia* L. (SA) fruit extracts against resistant Gram-positive UTI bacteria in companion animals by agar-well diffusion method; negative controls—mix of ethanol/distilled water (*v*/*v*) and DMSO; positive control—amoxicillin (center).

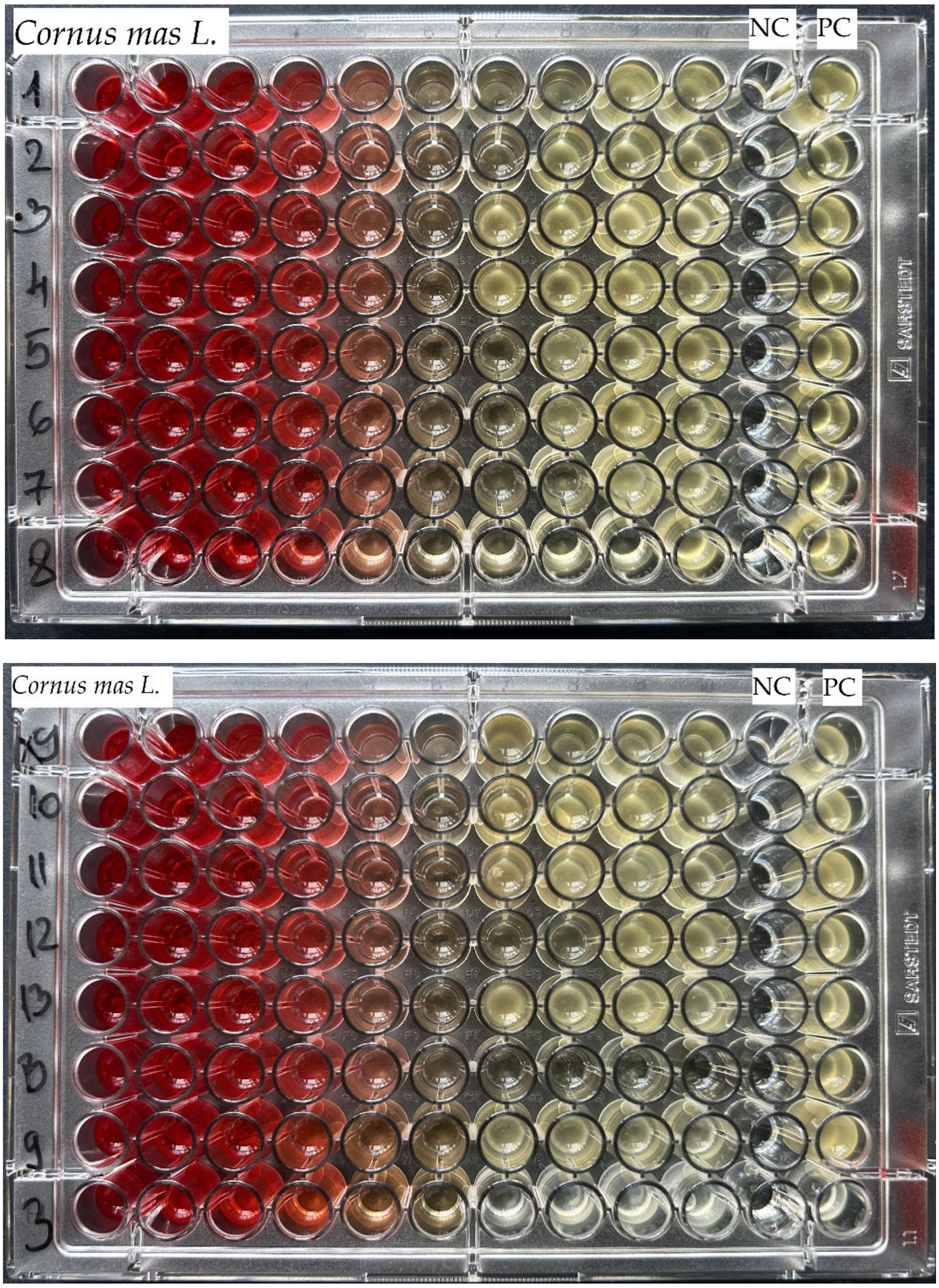

Figure S3. Antimicrobial activity of *Cornus mas* L. fruit extract against resistant UTI bacteria in companion animals by determining the minimum inhibitory concentration (MIC); NC—Negative control (mix of ethanol/distilled water *v*/*v*); PC—positive control (MH agar with bacterial suspension); 1—*E. coli* 612, 2—*E. coli* 531, 3—*P. mirabilis* 422, 4—*P. mirabilis* 582, 5—*K. pneumoniae*, 6—*K. oxytoca*, 7—*Ps. luteola*, 8—*E. faecalis*, 9—*E. faecium*, 10—*E. cloacae*, 11—*Ac. Baumannii*, 12—*S. lentus*, 13—*S. pseudintermedius*.

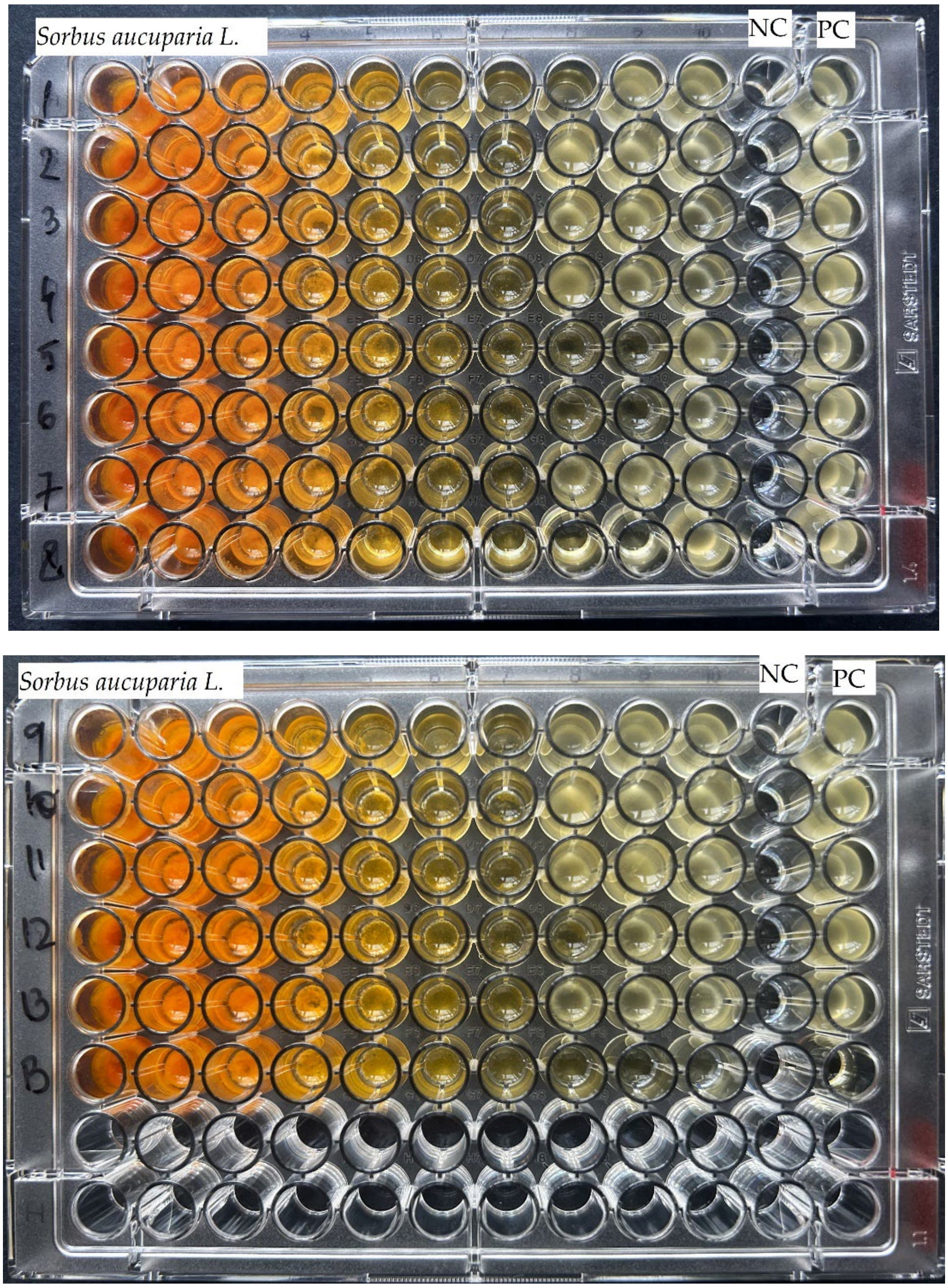

Figure S4. Antimicrobial activity of *Sorbus aucuparia* L. fruit extract against resistant UTI bacteria in companion animals by determining the minimum inhibitory concentration (MIC); NC—Negative control (mix of ethanol/distilled water *v*/*v*); PC—positive control (MH agar with bacterial suspension); 1—*E. coli* 612, 2—*E. coli* 531, 3—*P. mirabilis* 422, 4—*P. mirabilis* 582, 5—*K. pneumoniae*, 6—*K. oxytoca*, 7—*Ps. luteola*, 8—*E. faecalis*, 9—*E. faecium*, 10—*E. cloacae*, 11—*Ac. Baumannii*, 12—*S. lentus*, 13—*S. pseudintermedius*.
